# Eco-friendly biocontrol of the red palm weevil using the local brown alga *Hormophysa cuneiformis*

**DOI:** 10.1038/s41598-026-62701-1

**Published:** 2026-08-10

**Authors:** Nermin El Semary, Najla M. Albishi

**Affiliations:** Department of Biological Sciences, College of Science, King Faisal University, Al-Ahsa, 31982 Kingdom of Saudi Arabia

**Keywords:** *Hormophysa cuneiformis*, *Rhynchophorus ferrugineus*, Biological control, GC, FTIR, Pest Management, Biochemistry, Biotechnology, Ecology, Ecology, Environmental sciences, Microbiology, Plant sciences, Zoology

## Abstract

The red palm weevil (RPW), *Rhynchophorus ferrugineus* (Olivier) is a major destructive pest of date palms. The aqueous extract of a local brown alga was tested for the biocontrol of this insect. In that regard, we evaluated the insecticidal effects of aqueous extract of the brown alga, *Hormophysa cuneiformis*, against larvae and adults of *R. ferrugineus* through dietary bioassay. The FTIR of both the dried algal biomass and its aqueous extract was performed to reveal some of the functional groups present which are indicative of bioactive compounds. The insecticidal efficacy of its aqueous extract was assessed at three concentrations. Adult *R. ferrugineus* showed a concentration-dependent response to *H. cuneiformis* extract. After 10 days of exposure, mortality reached 80% at 10%, compared with 66.67% and 53.33% at 5% and 2.5%, respectively. Exposure of larval and pupal stages to the 5% aqueous extract significantly reduced survival, disrupted development, and impaired adult emergence, resulting in up to 68% larval mortality. In addition, the treatment significantly affected key biological traits, including larval weight and developmental duration of *R. ferrugineus*. The FTIR of the extract revealed the presence of functional groups related to broad range of metabolites and GC analysis showed diversity of compounds related to alcohols, secondary metabolites and other bioactive compounds which have insecticidal and larvicidal activities but are reported for the first time in this brown alga. This pest management strategy is both eco-friendly and cost-effective. The insecticidal effects observed show the potential application of *H. cuneiformis* extract as an eco-friendly biocontrol agent against *R. ferrugineus*. Algal aqueous extract can be easily supplied in irrigation water to plants, thereby deterring insects while being nutritious to plants.

## Introduction

The Red Palm Weevil (RPW), *Rhynchophorus ferrugineus* (Olivier) (Coleoptera: Dryophthoridae), is considered one of the most destructive insect pests affecting palm trees, particularly date palms (*Phoenix dactylifera*) across the Middle East, North Africa, Southern Europe, and parts of Asia^1,2^. A key factor for RPW invasion potential is its increased fertility and multivoltine nature, which produces multiple generations per year^3^. The RPW undergoes complete metamorphosis andspends the majority of its life cycle concealed within the tissues of its host^4^. The larvae burrow into the palm tree trunks and inner tissues, causing extensive damage that may remain unnoticed until the infestation becomes serious, ultimately resulting in the death of the tree^3,5,6^. Due to the hidden nature of its life cycle, control of *R. ferrugineus* remains a major challenge, prompting the exploration of alternative, environmentally friendly pest management strategies^7^. Traditional management approaches, including chemical insecticides and pheromone-based traps, have provided temporary relief but are increasingly criticized for their environmental impact, development of resistance, and limited efficacy against internal-feeding larval stages^8,9^. Additionally, the persistent use of synthetic pesticides poses a significant threat to non-target organisms and human health, emphasizing the urgent need for safer and more sustainable biocontrol options^3^. In this context, brown algae have emerged as promising sources of bioactive compounds with potent insecticidal, antifungal, and antimicrobial properties, highlighting their potential as effective and sustainable agents in pest management strategies^10–12^. Saber et al. demonstrated that all the algal and cyanobacterial treatments based on their crude extracts showed insecticidal activities against the cotton leafworm Spodoptera littoralis larvae, with 2nd larval instars being more susceptible. They also pointed out the scarcity of literature available on that approach^13^. Recently, Alfy et al. demonstrated that algal extracts induced substantial mortality in two economically important agricultural pests, the fall armyworm (*Spodoptera frugiperda*) and the root-knot nematode (*Meloidogyne incognita*)^14^. In addition, studies on brown algae such as *Sargassum* spp. and *Padina* spp. have reported promising larvicidal and ovicidal activities against various dipteran and coleopteran pests^15–17^. Refaay et al. pointed out that genera such as *Sargassum* (e.g., *Sargassum latifolium*) and *Jania rubens* are rich in phenolics and flavonoids that suppress pest immune responses. They also demonstrated that extracts of *Jania rubens* and *Colpomenia sinuosa* exhibited genotoxic and larvicidal activates against *Culex pipiens* larvae^18^. *Hormophysa cuneiformis* is an abundant brown alga widely distributed throughout the Arabian Gulf and known for its diverse biological activities^19^. However, despite its reported biological activities, the insecticidal potential of *H. cuneiformis* against agricultural insect pests, particularly *R. ferrugineus*, has not been investigated. Thus, this study aimed to evaluate the biocidal efficacy of the aqueous extract of *Hormophysa cuneiformis* against the larval and adult stages of *Rhynchophorus ferrugineus*. In addition, the chemical composition of the extract was characterized, and its insecticidal activity was evaluated to assess its potential as an environmentally friendly biocontrol agent. This eco-friendly and cost-effective approach has the potential to contribute to sustainable pest management, food security, and environmental sustainability.

## Materials and methods

### Preparation of algal extract

**Fig. 1:**
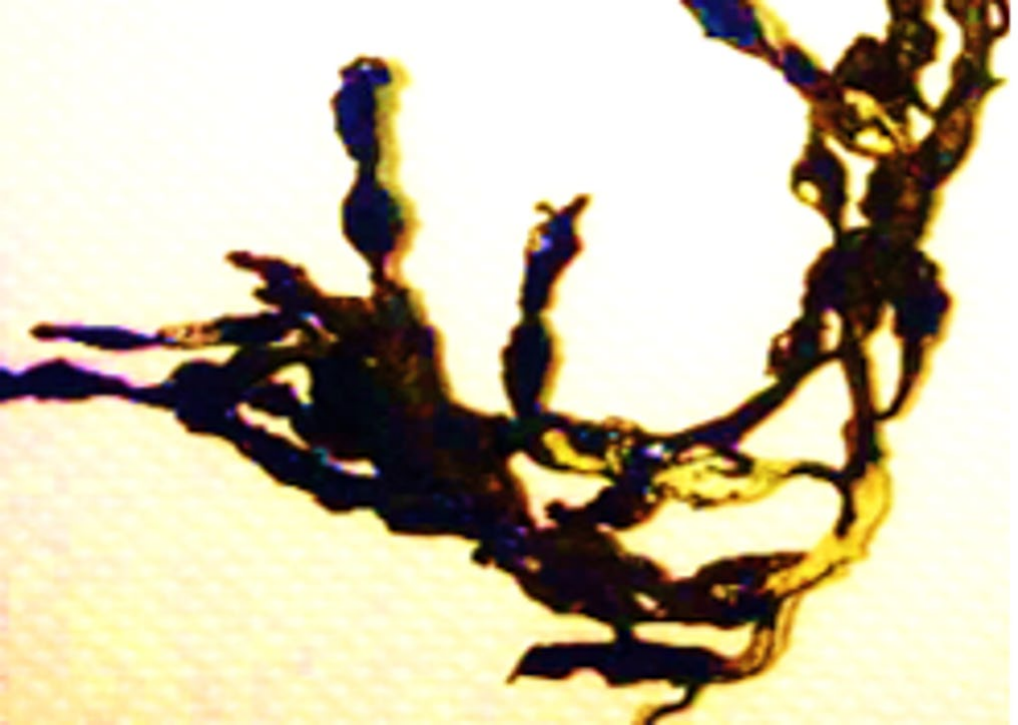
A photograph of the branched thallus of *Hormophysa cuneiformis* which is characterized by its rough surface and the presence of air vesicles.

The thalli of brown alga, *H. cuneiformis*, were collected in the Winter from AlUqair Beach, Saudi Arabia in February 2025. It is located approximately 70 km to 75 Km northeast of the city of Al-Hofuf, Al-Ahsa Governorate, Eastern Province, Kingdom of Saudi Arabia. The coordinates of the location are; Latitude: 25.6442°N; Longitude: 50.2150°E. The brown algae belong to Division: Phaeophyta; Class: Phaeophyceae; Order: Fucales; Family: Cystoseiraceae. *Hormophysa cuneiformis* (Fig. 1). The collected thalli were thoroughly washed with tap water followed by distilled water to remove adhering debris, sand, and salts, and then shade-dried at room temperature (25 °C) for 7 days. The thallus of *H. cuneiformis* ranges from 20 to 50 cm in length and is characterized by a tough, erect structure with regular branching and intercalary air vesicles^20^. The dried algal material was ground into a fine powder and soaked in distilled water at a 1:10 (w/v) ratio and stirred continuously for 24 h at ambient temperature. (25–28 °C). The mixture was filtered and stored at 4 °C until further use. The resulting extract served as the stock solution (10%, w/v). Working concentrations of 5% and 2.5% (w/v) were prepared by diluting the stock solution with distilled water. For the adult bioassays, concentrations of 2.5%, 5%, and 10% (w/v) were tested, whereas only the 5% concentration was used in the larval and pupal bioassays. All test solutions were freshly prepared prior to each bioassay against *R. ferrugineus*.

### Insect cultures

The red palm weevil, *R. ferrugineus*, was collected from naturally infested date palms from the village of Al-Qurain at 25°24′13″ N, 49°39′32″ E, altitude 165 m above sea level. It is situated to the west of Al-Ahsa Oasis, KSA. The weevil was reared under controlled laboratory conditions at 28 ± 1 °C, 75 ± 5% relative humidity (RH), and a 12:12 h (light: dark) photoperiod. For oviposition, adults were kept in ventilated plastic containers with fresh sugarcane pieces as both food source and oviposition substrate for adult *R. ferrugineus*, according to Aldawood et al.^21^. The newly hatched larvae were placed in groups of five per box into clean, small plastic boxes containing fresh sugarcane split sugarcane logs (10–15 cm) to provide access to the inner pulp and fibers for feeding and cocoon formation. Sugarcane was replaced every 3–5 days to prevent drying and frass accumulation. Larval development was assessed by monitoring head capsule molting and measuring larval size following the method described by El Shafie et al.^22^. After pupation, pupae were carefully transferred into clean containers lined with sterile paper and maintained under the same laboratory conditions until they emerged as adults. The newly emerged adults were then transferred into groups to separate containers for mating. The cycle was repeated to ensure a continuous supply of RPW for experimental purposes. Bioassays were performed on fifth-instar larvae, weighing 3.5–4 g, as well as adults, usually 5 days after ecdysis.

### Bioassay of the algal extract on red palm weevil

Bioassays were conducted by exposing RPW adults to aqueous algal extract at concentrations of 2.5%, 5%, and 10%. These concentrations were selected based on preliminary screening bioassays conducted to identify an appropriate range for evaluating the insecticidal activity of the extract against *R. ferrugineus*. These concentrations allowed the evaluation of dose-dependent effects while remaining practical and environmentally relevant. Control groups were treated with distilled water. Sugarcane pieces (10 cm in length) were added to each container after being pre-soaked in 10 mL of the aqueous extract for several minutes and air-dried on filter paper for 1 h before use. The treated food was placed in a plastic box. The box was capped with a cover that has a hole in it. In each treatment, groups of 10 adults were placed separately inside the plastic box containing three treated sugarcane pieces. The diet was replaced twice during the experiment period to maintain continuous exposure to the algal extract. Each treatment was replicated three times. During the experiment, mortality data from feeding on the treated sugarcane were recorded on days 3, 5, 7, and 10 after-treatments. The mortality was calculated by a formula:

$${\mathrm{Mortality}}~\left( \% \right) = {\frac{\text{Number of dead individuals}}{\text{Total number of individuals}}} \times 100$$

For larval bioassays, we evaluated the effect of a 5% aqueous algal extract on *R. ferrugineus* fifth instar larvae (5th instar) using the adult-treatment method described previously. A single concentration (5%) was selected for larval and pupal bioassays based on its demonstrated efficacy in preliminary evaluations and adult bioassays. The diet was replaced three times throughout the experiment to ensure continuous exposure to the algal extract. Larval body weights and mortality were recorded on days 3, 5, 10, and 15 after-treatments to assess the impact of the extract over time. For the pupal bioassays, newly formed pupae aged 48–72 h were collected from the laboratory colony. Only healthy pupae of similar size and developmental stage were selected to ensure uniformity and reduce experimental variation among treatments. Pupae were sprayed with the 5% aqueous extract, and their development was monitored. The bioassay was conducted using three replicates under the same laboratory conditions described previously. A concentration of 5% was selected for the larval and pupal bioassays based on preliminary screening results and its demonstrated biological activity. This concentration was used to evaluate the effects of the extract on immature developmental stages, including mortality, growth, and development, rather than to establish a dose–response relationship or estimate LC₅₀ values.

### FTIR analysis of dry biomass and aqueous extract of *H. cuneiformis*

Chemical functional groups were identified by the banding/stretching patterns in the FTIR spectrum. These functional groups are indicative of many bioactive compounds found in both dry biomass of *H. cuneiformis* and its aqueous extract. The FTIR spectra in the range of 800–4000 cm^− 1^ with automatic signal gain, scanning calorimetry (Agilent Technologies - Cary 630 FTIR, USA). The Agilent Technologies Cary 630 FTIR Spectrometer offers a resolution of ≤ 2 cm⁻¹ (less than or equal to 2 wavenumbers), a standard specification for this compact, versatile instrument used for rapid materials analysis, providing good spectral details. Chemical functional groups were identified by the banding/stretching patterns in the FTIR spectrum. These functional groups are indicative of many bioactive compounds found in both dry biomass of *H. cuneiformis* and its aqueous extract. The samples were tested in triplicates to ascertain the banding pattern.

### GC-mass analysis

The Gas chromatography mass spectroscopy was performed. Two grams of dry algal biomass were extracted in 10 mL Distilled water. The extract was left to stand for two days then 10 mL of Dicholromethane (pure Grade, Sigma, Germany) was added for extraction. This step is termed derivatization and was performed in order to allow volatilization of some of the non-volatile compounds as a pre-requisite for Gas Chromatography. The mixture was homogenized, then centrifuged at 6000 rpm for 10 min. The upper most layer was decanted and the lower most layer was re-centrifuged to ascertain purity of any contaminants. The GC-mass analysis was performed using a Shimadzu-QP-2010 S plus GC-MS instrument. One microliter (µL) of the sample solution containing 100 micrograms (µg) of extract per microliter (ul) was injected. The tool was fitted with a capillary column (Rtx-1 30 m ×0.32 mm I.D., 0.25 μm) and an autosampler autoinjector (AOC-20i + s). The oven’s temperature was adjusted to begin at 100 °C, maintain it there for three minutes, and then rise by 15 °C every minute to 220 °C. After that, it was raised to 250 °C at a pace of 15 °C per minute for one minute while being kept at 220 °C. For the course of five minutes, the temperature remained unchanged. The injector and mass interface temperatures are configured in split mode at 250 °C. The linear velocity in the column is 58.8 cm/sec due to the 2.50 ml/min flow rate of the Helium carrier gas. The ion source temperature was set at 210 °C, the solvent cut length was set to 3.0 min, and the compounds were acquired by scanning mode with the MS detector in EI-mode. Mass spectra were recorded in the 50–550 m/z range under EI ionization (70 eV) with an 8.0 min solvent delay, using BSTFA with trimethylchlorosilane as the silylating agent. These were the operating conditions used for mass spectral analysis. Chromatographic data was processed and integrated using LabSolutions software version 4.1. Compound identification was performed by comparing the obtained mass spectra with those available in the NIST 11 mass spectral library and with published reference data^23^. Chromatographic data were processed and integrated using LabSolutions software version 4.1 (Shimadzu Corporation, Kyoto, Japan; https://www.shimadzu.com). Compound identification was performed by comparison with the NIST 11 Mass spectral library (National Institute of Standards and Technology, Gaithersburg, MD, USA; https://www.nist.gov).

### Phytochemical screening

#### Test for phenols and phlorotannins

Approximately four drops of 10% ferric chloride were added to 1 ml of the extract. The development of dark black-brown coloration is indicative of a positive result for phenolic compounds, primarily phlorotannins in brown algae. In contrast, the absence of a color change, with the solution remaining pale yellow, is indicative of a negative result^24^.

#### Test for alginates

calcium chloride (CaCl₂) solution was added to the neutralized extract. The formation of a gelatinous or fibrous precipitate confirmed the presence of alginate^25^.

### Statistical analysis

Statistical analyses were performed using GraphPad Prism version 5 (GraphPad Software, San Diego, CA, USA; https://www.graphpad.com). Data are presented as mean ± standard deviation (SD). Differences between two groups were analyzed using Student’s t-test, whereas comparisons among multiple groups and exposure periods were performed using two-way analysis of variance (ANOVA), followed by Tukey’s multiple comparison test when appropriate. Mean differences and their corresponding 95% confidence intervals (95% CI) were calculated as part of the post hoc multiple comparison analysis. Statistical significance was considered at *p* ≤ 0.05.

## Results

**Fig. 2:**
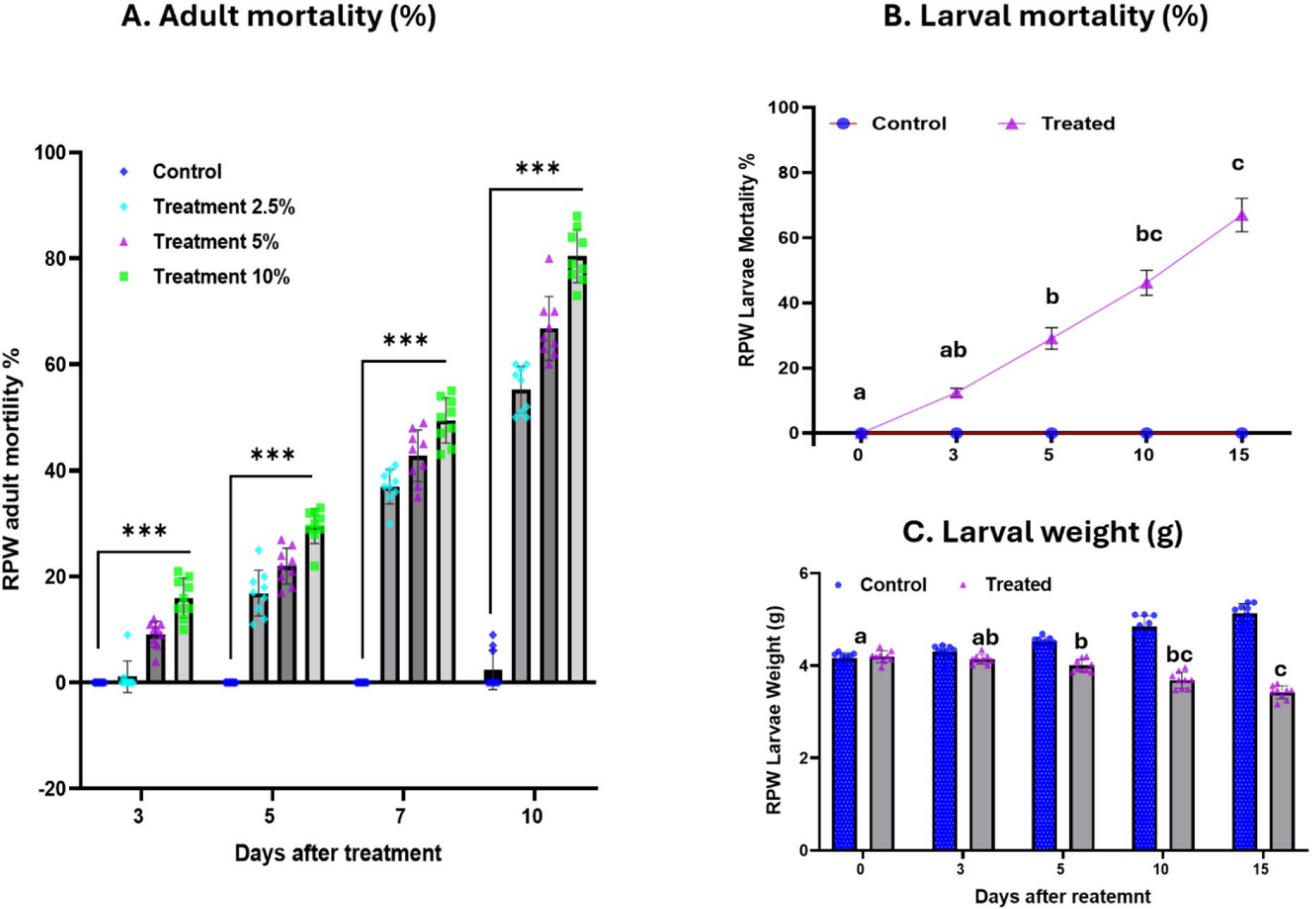
Effects of the aqueous extract of *Hormophysa cuneiformis* on adult and larva of *Rhynchophorus ferrugineus*. Data are presented as individual replicates with mean ± SE. Adult mortality data are presented as mean ± SD. Asterisks indicate statistically significant differences (*p* < 0.001, two-way ANOVA).

Adult mortality of RPW increased progressively with both time and concentration of the aqueous extract of *H. cuneiformis* (Fig. 2-A). Two-way ANOVA revealed a highly significant effect of time (F₃,₁₃₇ = 129.4, *p* < 0.0001) and concentration (F₃,₁₃₇ = 120.5, *p* < 0.0001) on adult mortality. At 3 days after-treatment, mortality ranged from 0% (control and 2.5%) to 16.67% at 10%. By day 5, the mortality reached 30% at the 10% concentration. Significant increases in mortality were observed in all treated groups on days 7 and 10. The highest. The highest concentration 10% resulted in 80% mortality by day 10, while the control group had only 2.0% mortality. In addition, fifth-instar larvae of *R. ferrugineus* were fed sugarcane pieces treated with 5% aqueous algal extract over 15 days. The treated group exhibited a sharp increase in mortality starting from day 5, while the control group had 0% mortality (Fig. 2-B). Larval mortality increased progressively throughout the experimental period, reaching 68% by day 15 after treatment. Statistical analysis confirmed significant differences (*p* < 0.05) between the groups from day 5 onward. Figure 2-C illustrates changes in the mean larval weight of *R. ferrugineus* over time in the control and algae-treated groups. The Larval weight was significantly affected by the aqueous extract of *H. cuneiformis* over the observation period. In the early days, no significant differences were observed on day 3, but from day 5 onward, the treated group showed a marked suppression of weight gain. The mean weight of RPW larvae in the control group increased steadily, reaching 5.24 ± 0.03 g by day 15. The mean weight of RPW larvae in the control group increased steadily, reaching 5.24 ± 0.03 g by day 15. In contrast, treated larvae showed significantly reduced weight gain, reaching only 3.47 ± 0.21 g by day 15. The estimated mean difference between the control and treated groups was 0.707 g (95% CI: 0.5534–0.8614), indicating a significant reduction in larval weight following treatment. Two-way ANOVA revealed a significant treatment effect on larval weight (F₁,₈₄ = 83.47, *p* < 0.0001).

**Fig. 3:**
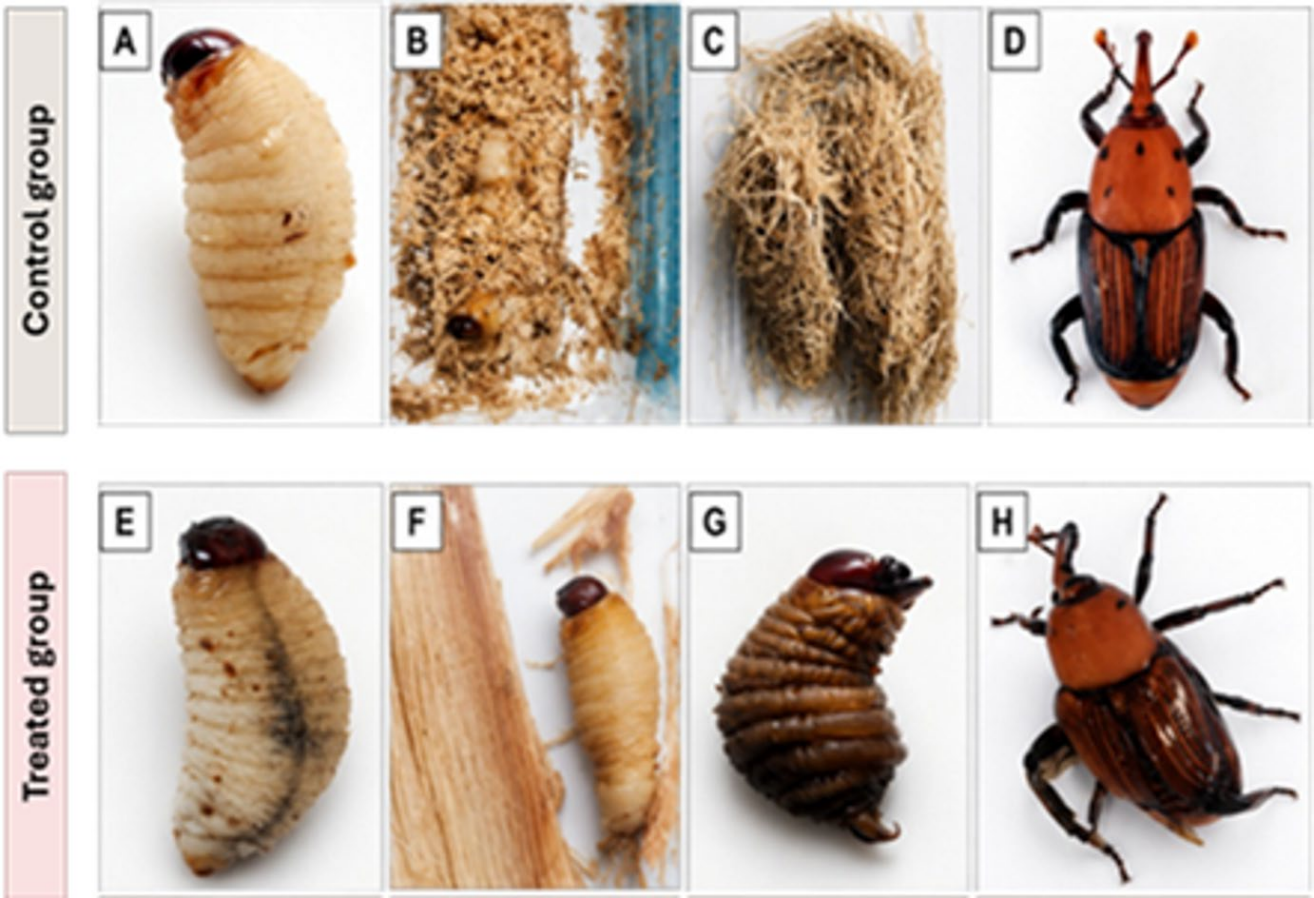
Phenotypic characteristics of *Rhynchophorus ferrugineus* in the control and treated groups. (**A**–**D**) Normal development in the control group, including a healthy larva, feeding larva, cocoon formation, and normal adult emergence. (**E**–**G**) Developmental alterations following larval treatment with the aqueous extract of *Hormophysa cuneiformis*, including reduced growth and failure to pupate. (**H**) Abnormal adult emerging from a treated pupa.

Control larvae shown in Fig. 3-A appeared larger, more elongated, and pale cream-colored indicating that they were likely late instar larvae approaching the pupation stage. A few days later, larvae bored deeper into sugarcane stalks as they grew. Initially, larvae fed on softer tissues near the stalk’s surface, creating feeding tunnels (Fig. 3-B). Mature larvae subsequently pupated within the tunnel itself, often near its end, constructing a cocoon from chewed fibers and frass that lines the pupation chamber. Control larvae successfully pupated by day 15, developing into normal pupae and subsequently emerging as normal, fully formed, healthy adults (Fig. 3-C). In contrast, treated larvae were noticeably smaller with a darker, more wrinkled appearance and dehydrated compared to healthy controls (shown in Fig. 3-E). At day 15, no pupation was observed in the treated group, as most larvae experienced continuous weight loss and died before reaching the pupal stage (Fig. 3-G). Pupa sprayed with *H. cuneiformis* extract developed noticeable abnormalities during adult emerging compared with the control group. Treated pupae displayed reduced activity, malformed wings, incomplete body hardening, and deformities in the thoracic or abdominal segments (Fig. 3-H). In some cases, adults failed to emerge completely from the pupal case or showed post-emergence. The control pupae developed into normal, fully formed adults (see Fig. 3-D).

**Fig. 4:**
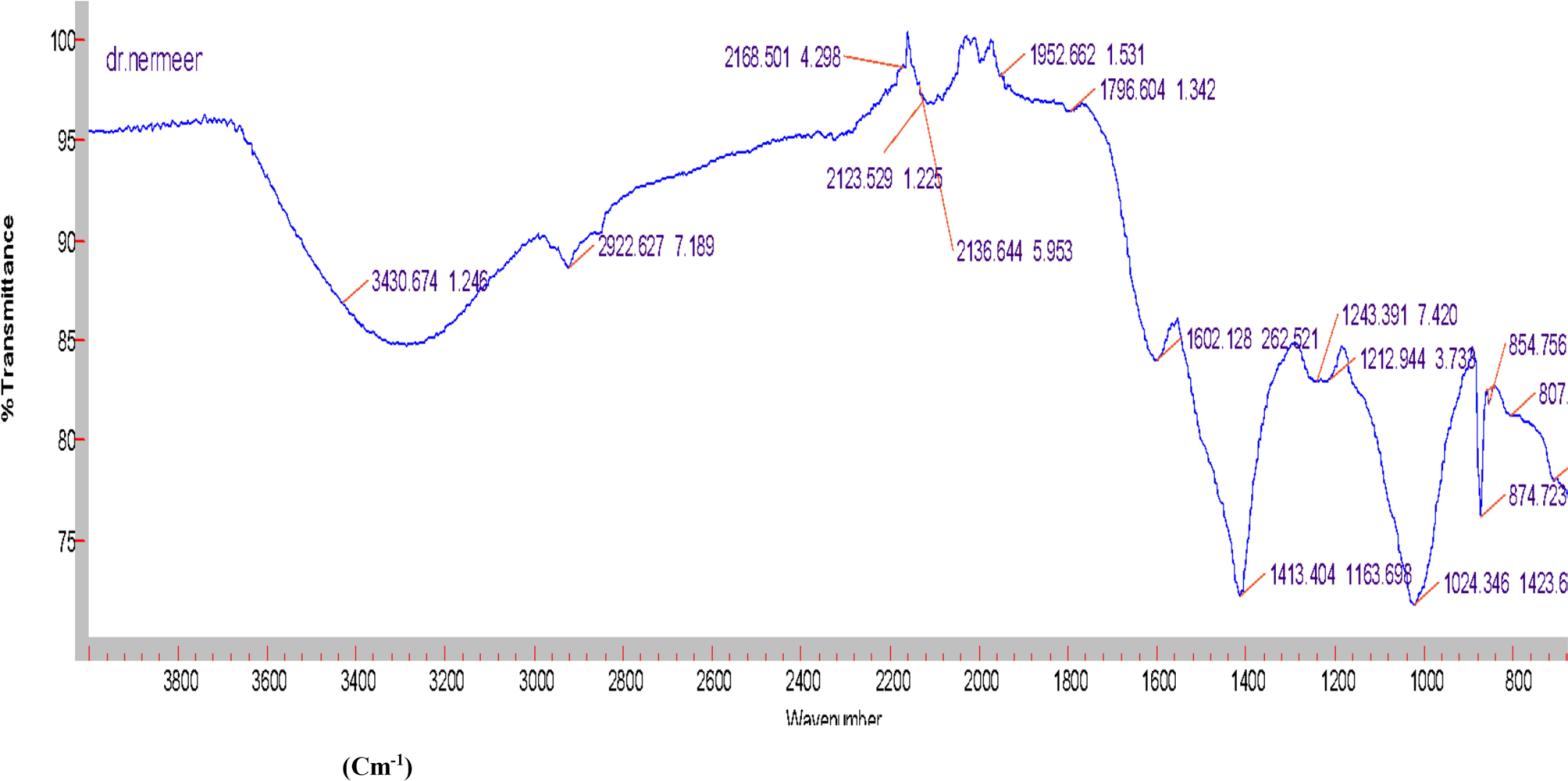
FTIR of the air-dried biomass of *Hormophysa cuneiformis*.

**Fig. 5:**
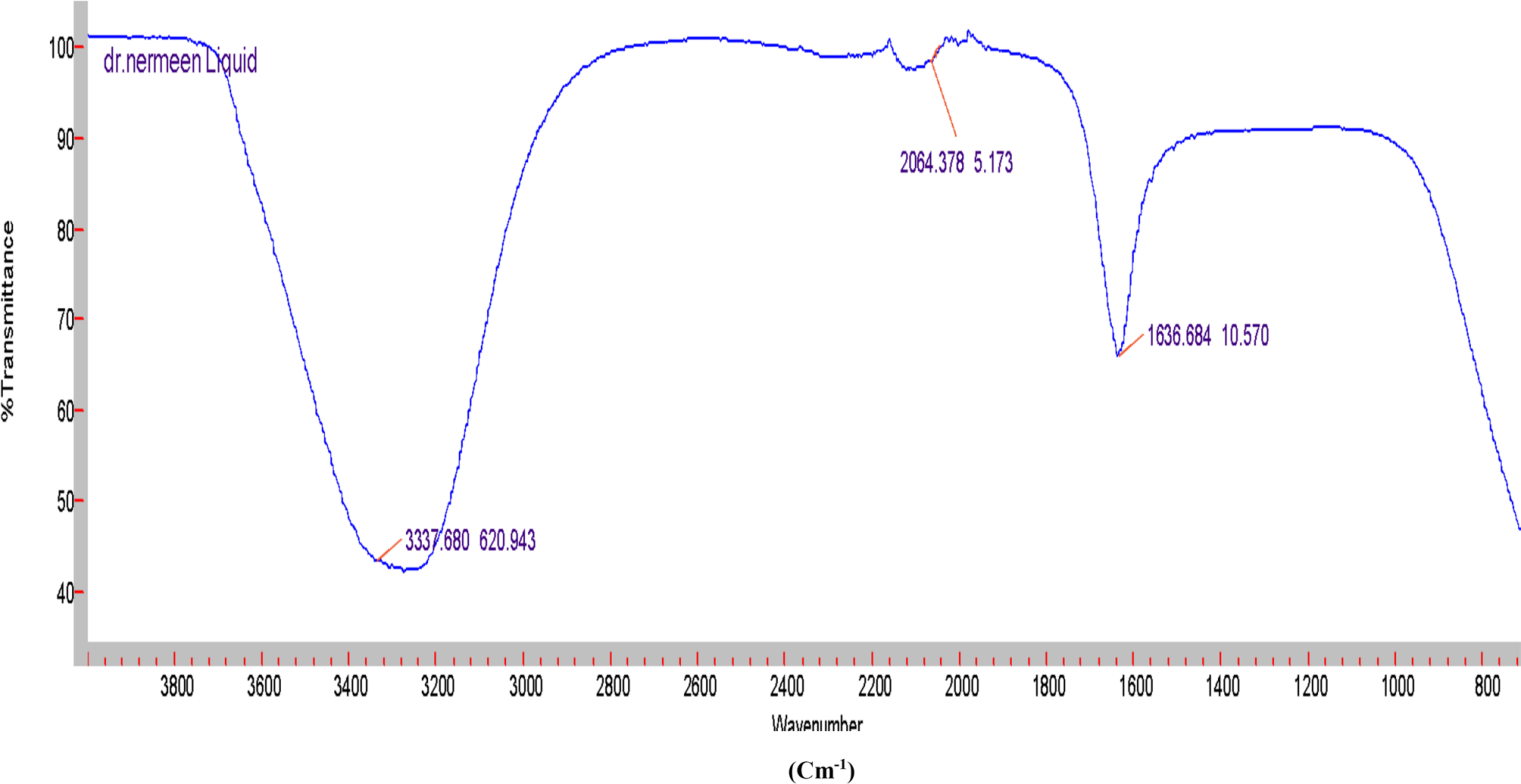
FTIR of the aqueous extract of *Hormophysa cuneiformis*. The aqueous extraction was performed on air-dried algal biomass.

The FTIR spectroscopy on the air-dried biomass of the alga *H. cuneiformis* revealed the presence of numerous functional groups indicative of many bioactive and growth-related compounds (Fig. 4). The identification of functional groups based on banding patterns^43^. The -OH broad band stretching in the range between 3600 and 3400 cm⁻¹ wavenumber which can be indicative of many compounds including mannitol whose presence is confirmed by finding C–O near 1300–1000 cm⁻¹. The presence of both OH group and the aromatic C–O–C ether bond can be suggestive of phlorotannin^44,45^. Aromatic C = C bonds can be seen in the ~ 1600–1500 cm⁻¹ region. Also, N–H band in the range of 3500–3100 cm⁻¹ wavenumber. The medium to strong absorptions in the region 1600–1450 cm⁻¹ strongly indicates aromatic ring. Presence of aromatic ketones with a characteristic C = O functional group with phenyl ring is indicated from absorption in the range of 1700–1680 cm⁻¹ for C = O and 1600–1450 cm⁻¹ for ring. In addition, sulphated polysaccharides exhibited a broad band around 1220–1260 cm⁻¹ indicative of the presence of sulphate ester groups^45^. Moreover, C–H stretching around 3000 –2000 cm⁻¹, and strong peaks for asymmetric and symmetric COO⁻ carboxylate stretching at around 1635 cm⁻¹ and 1410 cm⁻¹ respectively. Peak detected near 1035 cm⁻¹ represents band of C–O–C stretching vibrations of the glycosidic bridges^46^. Halogens such as Cl are indicated by stretch in the range of 800 –600 cm^− 1^. Aqueous extract of the air-dried biomass of *H. cuneiformis* showed characteristic banding and absorption of OH, C-O stretching, C-H stretching. In addition, banding patterns characteristic of the ether, aromatic ring, sulfur, carboxylate are all present (Fig. 5).

GC–MS analysis detected a diverse range of derivatized metabolites. The detected constituents included mannitol derivatives, halogenated compounds, sulfur-containing compounds, alcohols, phenolic derivatives, terpene-like compounds, sterol derivatives, and other bioactive metabolites. The identified compounds, together with their retention times (RT), relative peak areas, molecular formulas, and proposed biocontrol activities, are summarized in Table 1. Phytochemical screening revealed a positive reaction for polyphenols and tannins, as evidenced by the color change following the addition of ferric chloride (FeCl₃). The alginate assay also yielded a positive result, indicated by the formation of a gelatinous precipitate following the addition of calcium chloride (CaCl₂).

**Table 1:** Major compounds identified in the aqueous extract of *Hormophysa cuneiformis* using GC–MS analysis and their previously reported biological activities related to insecticidal, larvicidal, repellent, or biocontrol effects as documented in the literature.

Compounds (RT, Peak Area %)	Retention time	Area%	Molecular formula	Potential relevance to insecticidal and/or larvicidal activity
2-Pyrrolidinecarboxylic acid- 5-oxo-, ethyl ester	6.349	0.05%	C_7_H_11_NO_3_	Amino acid derivative found in both microalgae and macroalgae (seaweed). The pyrrole ring structure within the molecule is similar to other naturally occurring pyrrole derivatives that exhibit significant toxicity against pests like the cotton leafworm ^26^
1,2,3,4,5,6-Hexakis-O-(trimethylsilyl)-d-mannitol	11.217	0.48%	C_24_H_62_O_6_Si_6_	Mannitol derivative (osmotic and digestive entomological toxin ^27,28^
Benzeneacetic acid, alpha.,3,4-tris[(trimethylsilyl)oxy]-, trimethylsilyl ester	12.375	3.87%	C_20_H_40_ O_5_Si_4_	polyphenolic flavonoidal derivative with reported larvicidal activity ^29^
Cyclononasiloxane, octadecamethyl-	15.88	11.3%	C_18_H_54_O_9_S_9_	Cyclic Siloxane (Polysiloxane) (reported in a biocontrol study ^30^
3-Chloropropionic acid, heptadecyl ester	16	0.25%	C_20_H_39_ClO_2_	Halogen-derivative (acting as larvicides, spatial repellents, and enzyme inhibitors (e.g., inhibiting ache) against harmful pests ^31^
Sulfurous acid, octadecyl 2-propyl ester	16.71	0.18%	C_21_H_44_O_3_S	Sulfur-derivative (reported in a study on larvicidal activity of plant extracts ^32^
1-Heptatriacotanol	16.94	1.39%	C_37_H_76_O	Long chain alcohol that is frequently identified during GC-MS (Gas Chromatography-Mass Spectrometry) analysis of plant extracts that successfully deter or kill common agricultural and stored-product pests ^33^
Cholest-22-ene-21-ol, 3,5-dehydro-6-methoxy-, pivalate	17. 378	4.69%	C_33_H_54_O_3_	Sterol derivative with insecticidal activity. Research shows it acts as a potent, natural bioinsecticide ^34^
9,9-Dimethoxybicyclo[3.3.1]nona-2,4-dione	18.05	1.77%	C_11_H_16_O_4_	Volatile secondary metabolite frequently identified in marine algae extracts (like brown macroalgae) it acts as major component in biopesticide ^15^
3,7,11,15-Tetramethyl-2-hexadecen-1-ol	18.63	8.49%	C_20_H_40_O	Alcohol, which is widely known as phytol, an acyclic diterpene alcohol found naturally in chlorophyll. Extensive research confirms its insecticidal, larvicidal, and repellent activities against various pests ^35^
17-Pentatriacontene RT: 20.471-; Area 4.29%	20.471	4.29%	C₃₅H₇₀	A long-chain alkene identified in various macroalgae anti-inflammatory, anti-arthritic, and antimicrobial activities, contact toxicity and larvicidal activity against disease vectors and agricultural pests ^36^. Extracts containing this compound show promising results in deterring stored-product pests, such as the red flour beetle and the lesser grain borer ^37^
Erythro-9,10-Dibromopentacosane	21.154	10.57%	C₂₅H₅₀Br₂	a halogenated long-chain hydrocarbon. Found as a naturally-occurring secondary metabolite found in marine algae. with regard to insect biocontrol& larvicidal properties, halogenated compounds target insect nervous systems and developmental stages, making them potent agents against disease-vector such as mosquitoes ^38^
Piperidin-2-one, 6-(4-benzyloxy-3-methoxyphenyl)-5-nitro-	22.815	17.14%	C₁₉H₂₀N₂O₅	Similar piperin analogs are used in biopesticide ^39^
6.beta.-Pentylandrostane-3.beta.,5.alpha.,17.beta.-triol	23.144	11.25%	C₂₄H₄₂O₃	A steroidal compound that may help in through Neurotoxicity, Antifeedant activity, Repellency and olfactory disruption ^16,40,41^
2,2-Dimethyl-6-methylene-1-[3,5-dihydroxy-1-pentenyl]cyclohexan-1-perhydrol	24.572	0.2%	C_14_H_24_O_4_	Oxygenated terpene and secondary metabolite found in various plant and seaweed extracts. It is primarily known as a volatile phytochemical that is tested against mosquito larvae (such as *Culex pipiens* or *Aedes aegypti*) to evaluate eco-friendly larvicidal activity ^42^

## Discussion

The present study highlights the bio-insecticidal potential of the brown alga *H. cuneiformis* against various developmental stages of the red palm weevil, *R. ferrugineus*, a notorious pest of date palms worldwide. The aqueous extract exhibited significant toxicity against both larval and adult stages, with mortality increasing in a concentration- and time-dependent manner. Similar findings have been reported for other natural products. For example, extracts of *Thymus vulgaris* and *Ocimum basilicum* have demonstrated bio-insecticidal activity against *R. ferrugineus*, supporting the potential of plant-derived compounds as environmentally friendly alternatives for the management of this economically important pest^47^. These findings support the potential use of marine algae-based products in integrated pest management (IPM) programs. The efficacy of *H. cuneiformis* extract, particularly at 10% concentration, where adult mortality reached 80% by day 10, is notable and compares favorably with earlier studies involving other marine macroalgae. Thus, aqueous algal extract indeed acts as a bio-insecticide, particularly with increased exposure time. Concomitantly, *Sargassum* spp. and *Padina* spp. extracts have previously shown larvicidal effects against mosquito pests^45,48–50^. In addition, brown algal-derived products have demonstrated insecticidal activity against a wide range of insect pests, including agricultural species such as *Diaphorina citri*, *Dysdercus cingulatus*, *Aphis craccivora*, *Plutella xylostella*, and *Spodoptera littoralis*, as well as coleopteran pests such as *Callosobruchus maculatus*^51,52^. Collectively, these findings highlight the presence of diverse bioactive metabolites in brown algae and support their potential as environmentally friendly agents for sustainable insect pest management. The inhibition of larval development, and the prevention of pupation observed in our study further emphasizes the disruptive effects of *H. cuneiformis* on RPW physiology and metamorphosis. Indeed, by days 10 and 15, the growth reduction was highly significant, indicating a strong cumulative effect of the treatment on larval fitness and development. These results suggest that the algal extract disrupted the normal development and survival of RPW, indicating its potential as a biocontrol agent.

The observed bioactivity may be attributed to the presence of diverse secondary metabolites, including phenylpropanoids, polyphenols, amino acids, and esters, which have been documented to interfere with insect hormonal regulation, digestion, and neural transmission^53,54^. These compounds are likely to act synergistically to cause lethal and sub-lethal effects, such as reduced weight gain, altered development, and reproductive suppression. These bioactivities are largely attributed to the presence of phlorotannin, fucoidans, and halogenated compounds, which interfere with vital physiological processes in pests and pathogens^55^.

This aligns with previous findings that marine algal extracts can influence insect behavior through neuroactive or deterrent compounds^11,17^. They examined the aqueous extract and hydrolats from *Sargassum* species and found that they could deter oviposition, induce contact irritancy, and modify blood-feeding behavior in female *Aedes aegypti*, despite moderate direct toxicity. Moreover, the morphological abnormalities observed in both larvae and emergent adults, such as incomplete sclerotization, malformed cocoons, and deformed wings, further demonstrate the extract’s interference with the weevil’s developmental processes. These phenotypic outcomes are consistent with the hypothesis that algal bioactive compounds disrupt endocrine or molting-related pathways in insects^56^. With regard to *H. cuneiformis* which is also rich in other bioactive compounds like fatty acids and their esters as well as the presence of phenolics, flavonoids, and tannins, clearly indicates algal biocontrol potential. These compounds are also responsible for its observed antimicrobial and anticancer properties. The presence of the sulfur compound was noted as a minor component, with its quantity potentially influenced by the algal collection period^57^. Studies have found that extracts of *H. cuneiformis* are rich in these compounds, which are known for their anti-inflammatory, antimicrobial compounds and antioxidant activity. In addition, it has been reported that several types of extracts including ethanolic, acidified ethanolic and aqueous extracts of the marine brown alga *H. cuneiformis* have shown antioxidant activity. This particular alga is found in abundance along the shores of the Red Sea and Arabian Gulf. A thorough investigation reported high reducing power as indicated by DPPH radical scavenging assay, high total flavonoid content, high total antioxidant capacity, and high total polyphenol content which are all regarded as indicators of the antioxidant properties of *H. cuneiformis*^58^. In addition, they analyzed its nutritional components and found it to be rich in fiber (34.24%), ash (30.93%), carbohydrates (28.5%), and proteins (5.24%), with low lipid content (1.09%) on dry weight. The alga is also known for its multiple bioactive compounds of different activities against other organisms. For example, Salem et al. reported its antibacterial activity^59^ whereas Mohamed and Saber showed its antifungal activity^60^.

The chemical analyses including FTIR, GC-mass and phytochemical screening confirmed the richness of that alga with bioactive compounds that have insecticidal/larvicidal impact with diverse modes of action. The GC revealed the presence of many compounds. However, those identified are naturally volatile or can be derivatized. This comes from the fact that gas chromatography operates on gaseous state of the matter, and some cannot be derivatised and so partial identification for some of the compounds, but not all compounds, is feasible. That is why the integration of both FTIR and GC-mass data as well as phytochemical screening is highly important. Alginates, for example, are anionic soluble polysaccharides with the characteristic FTIR which includes a broad band for O–H stretching, C–H stretching around 2920–2850 cm⁻¹, and strong peaks for asymmetric and symmetric COO⁻ carboxylate stretching at around 1635 cm⁻¹ and 1410 cm⁻¹ respectively^25,43^. Alginates do not have a single, fixed GC mass; instead, their molecular mass is usually expressed as a broad average range determined via Gel Permeation Chromatography^46^. Another example is the phlorotannin which are complex polyphenols found exclusively in brown algae, built from polymerized phloroglucinol units (1,3,5-hydroxybenzene). They range in mass from (126) Da (monomer) to over (650) kDa. They identified several bioactive compounds including phloroglucinol, eckol, fucodiphloroethol G and phlorofucofuroeckol A^45^. Liquid Chromatography-Mass Spectrometry (LC-MS) is the most accurate analytical method^46^. Phlorotannins have both soluble and insoluble (cell-wall-bound) forms, with soluble phlorotannins contained within intracellular vesicles called physodes, while cell-wall-bound phlorotannins are insoluble and integrated into the cell wall. Their solubility varies based on structure, concentration, and environmental factors, with the soluble form often playing a more dominant ecological role. Phlorotannins are also involved in deterring herbivores and protecting them from UV radiation.

These findings suggest that *H. cuneiformis* may harbor similar or even superior bioactive properties suitable for integrated pest management (IPM) programs targeting *R. ferrugineus*. Moreover, the ecological abundance and renewable nature of brown algae, coupled with their biodegradability and low toxicity to non-target organisms, position them as ideal candidates for sustainable pest control solutions^61^. Given the rising demand for eco-friendly biopesticides and the limitations of chemical control, this study emphasized the pesticidal and physiological effects of *H. cuneiformis* on *R. ferrugineus*. These findings also indicated that the algal extract treatment not only suppressed larval growth but also completely inhibited progression to the pupal stage. Regarding the presence of some sulphated polysaccharides in the aqueous extract is not surprising as some fucoidans (a type of sulphated polysaccharides) are water-soluble^62^. However, the solubility of sulfated polysaccharide is dependent on its specific structure, molecular weight, the sulfation degree, and the cations present. Fucoidans when extracted will be extracted as fucans which are water-soluble^63^. Phlorotannins and sulfated polysaccharides, especially those from marine seaweeds, show promise as natural insecticides due to their antimicrobial, antifungal, and membrane-damaging properties. These seaweed-derived compounds are biodegradable, have low toxicity to beneficial organisms, and offer a sustainable alternative to synthetic pesticides. Indeed, five closely related species of seaweed were utilized in the biocontrol of *Anopheles stephensi*,* Aedes aegypti*,* and Culex quinquefasciatus*^64^. Mannitol, a water- soluble alcohol widely found in brown algae acts in the biocontrol of insects by disrupting their osmoregulation and carbohydrate digestion. Thus, mannitol acts as osmotic and digestive toxin resulting in a concentration-dependent mortality and stunting larval growth as well as limiting adult lifespan in species that readily consume as in certain insects such as *Drosophila* fruit flies^27^.

The phytochemical analysis was positive for Phenol and phlorotannin^65^ as well as alginates. Alginates are anionic polysaccharides composed of mannuronic acid and guluronic acid are soluble in case of sodium alginate but insoluble in case of calcium alginate. The FTIR further reinforced this as they have a broad band for O–H stretching, C–H stretching, and strong peaks for asymmetric and symmetric COO⁻ carboxylate stretching. Mannitol is also present in the aqueous extract and dry matter alike. Mannitol is a significant sugar alcohol, or polyol, found in brown algae. It serves as a key photosynthetic end product, acting as a primary form of carbon storage, an osmo-protectant to protect against osmotic stress, and an antioxidant. These roles enable brown algae to survive in fluctuating marine environments, such as intertidal zones. Mannitol is a major component of brown algae’s dry weight and is commercially extracted for its uses in the food, chemical and pharmaceutical industries^66^. From an ecological perspective, the use of *H. cuneiformis* offers a sustainable and environmentally benign alternative to synthetic pesticides. These outcomes align with concerns regarding non-target toxicity, resistance development, and environmental contamination often associated with synthetic insecticides^67^, which botanical alternatives aim to mitigate^68^. Similar effects from other brown algae were reported^15–17,69−71^. Indeed, the biodegradability, abundance, and renewability of marine macroalgae add further value to their integration into pest management systems. The results support the potential of algal-based formulations in sustainable pest management strategies. However, while the results are promising, further research is required to determine the field efficacy, optimal formulation, and potential non-target effects of *H. cuneiformis* extracts. Investigations into the extract’s mode of action at molecular and biochemical levels will also provide deeper insight into its insecticidal mechanisms. However, Sekar et al. listed some of the challenges that may face the use of seaweed as insecticide such as scalability and extraction maximization, they emphasized the use of advanced approaches and extraction technologies to overcome these challenges 72. The alga proved to be a prolific source of many compounds as evidenced from FTIR. The extraction of these compounds most likely will achieve maximum insecticidal and insect-deterrent effects while providing useful nutrients for the palm plant.

The GC mass confirmed the presence of many bioactive compounds with reported insecticidal and/or larvicidal activities. Marine algae produce various secondary metabolites—such as steroids, terpenes, and polysaccharides—to defend themselves against predators and pests in the harsh marine environment target insects through the following primary mechanisms^16,40^. These include acting on several levels namely;

- Neurotoxicity: Many algal metabolites (particularly certain terpenes and alkaloids) disrupt the insect nervous system, causing paralysis or rapid mortality.
- Antifeedant Activity: Compounds alter the insect’s taste receptors or induce toxicity upon ingestion, causing pests to cease feeding and eventually starve.
- Repellency: Volatile secondary metabolites deter insects from landing, feeding, or laying eggs on treated crops or surfaces.
- Growth and Development Regulation: Extracts can disrupt the insect life cycle by slowing larval development, preventing eggs from hatching, or blocking chitin production (which inhibits proper exoskeleton formation).
- Olfactory Disruption: Specific compounds interfere with the antenna^41^.

A suggested mode of action involves inducing neurotoxic effects of secondary metabolites such as terpenes, flavonoids, saponins and their derivatives which may inhibit acetylcholinesterase. Thereby, causing an accumulation of acetylcholine. Meanwhile, glutathione, which is a major main cofactor for glutathione S-transferase (GST), was significantly depleted leading to oxidative stress which resulted from excessive Reactive Oxygen Species production triggered by algal; bioactive metabolites that impair mitochondrial function^31^. Another suggested mechanism is that the compounds of the extract inhibited chitin (exoskeleton) synthesis is through inhibiting with regard to secondary metabolites’ insecticidal mode of action, the compound **2**,**2-**Dimethyl-6-methylene-1-[3,5-dihydroxy-1-pentenyl] cyclohexan-1-perhydrol for example is a naturally occurring volatile compound in plant extracts. It can result in toxicity where it can cause mortality in target pests by acting on their nervous system, often via competitive inhibition of enzymes such as Acetylcholinesterase. It also acts as antifeedant where it discourages insects from feeding on treated plants or stored crops. Finally, it can act as repellent thereby reducing oviposition (egg-laying)^71^.

This biocontrol strategy provides an effective non-harmful alternative to the harmful chemical insecticides that usually leave residuals on the plant causing serious health effects and pollution of the environment. Nonetheless, the rationale for using aqueous extraction was based on both practical and ecological considerations. Aqueous extracts are environmentally safe, cost-effective, and more stable for large scale agriculture applications, particularly in palm trees as they can be added to irrigation water or even left in soil. Indeed, aqueous extraction mimics natural leaching processes in soil and irrigation systems, allowing bioactive metabolites to be readily available for root uptake. Several biologically active algal compounds are water-soluble and have been reported to exhibit insecticidal and bio-stimulant activities^16,42,65,72–76^. The focus of this study was to evaluate the feasibility for field application rather than exhaustive extraction of all secondary metabolites. Moreover, we practically elucidated by FTIR and GC-mass analysis that there are several bioactive compounds with insecticidal/larvicidal activities from previous studies, none of which was on our algal type, hence the study is novel in revealing the presence and activity of these compounds in *Hormophysa cuneiformis*. The response of the insect‘s different stages to the extract shows its positive biocontrol impact. In addition, we wanted to present a cost-effective protocol that even farmers can use with no expensive organic solvents and their toxic residuals after extraction.

## Conclusion

The aqueous extract of the brown alga *Hormophysa cuneiformis* demonstrated promising biocidal activity against both the larval and adult stages of *Rhynchophorus ferrugineus*. FTIR analysis confirmed the presence of several bioactive functional groups that may contribute to the observed insecticidal effects. The extract significantly reduced insect survival and adversely affected development, highlighting its potential as an environmentally friendly and sustainable alternative for RPW management. Moreover, the use of a locally abundant and renewable marine resource in combination with a simple aqueous extraction method enhances the practicality, cost-effectiveness, and environmental sustainability of this biocontrol strategy. Nevertheless, the present study was conducted exclusively under laboratory conditions, and the efficacy of the extract under field conditions remains to be established. Furthermore, the specific bioactive compounds responsible for the observed insecticidal activity and their underlying mechanisms of action were only indicated but were not separated individually and investigated. Future research should focus on the isolation and characterization of the active constituents, elucidation of their modes of action, assessment of their environmental safety and effects on non-target organisms and validation of their efficacy through field trials. Such investigations will be essential for supporting the development of *H. cuneiformis-*based biopesticides for the sustainable management of *R. ferrugineus.*

## Data Availability

All data generated in the research are provided in the manuscript.

## References

[1] Hoddle, M. S. et al. How Far Can the Red Palm Weevil (Coleoptera: Curculionidae) Fly? Computerized Flight Mill Studies With Field-Captured Weevils. *J. Econ. Entomol.* **108**, 2599–2609. 10.1093/jee/tov240 (2015). 26470385 10.1093/jee/tov240

[2] Sutanto, K. D. et al. An overview of evaluating the efficacy of microbial pathogens for biological control of the red palm weevil *Rhynchophorus ferrugineus* (Olivier, 1790) (Coleoptera: Curculionidae) insights from laboratory and field studies. *Egypt. J. Biol. Pest Control*. **35** (3). 10.1186/s41938-025-00839-2 (2025).

[3] Mohammed, M. E. A., El-Shafie, H. A. & Alhajhoj, M. Recent Trends in the Early Detection of the Invasive Red Palm Weevil, *Rhynchophorus ferrugineus* (Olivier). In *Invasive Species - Introduction Pathways, Economic Impact, and Possible Management Options*. IntechOpen (2020). 10.5772/intechopen.93393

[4] Manee, M. M., Alqahtani, F. H., Al-Shomrani, B. M., El-Shafie, H. A. F. & Dias, G. B. Omics in the Red Palm Weevil *Rhynchophorus ferrugineus* (Olivier) (Coleoptera: Curculionidae): A Bridge to the Pest. *Insects* **14**10.3390/insects14030255 (2023). 10.3390/insects14030255 PMC10054242 36975940

[5] Giblin-Davis, R., Faleiro, J. R., Jaques, J., Pena, J. E. & Vidyasagar, P. Biology and Management of the Red Palm Weevil, *Rhynchophorus ferrugineus*. *Potential. Invasive Pests Agric. Crop*. **3**, 1. 10.13140/2.1.1029.1202 (2013).

[6] Tagliavia, M., Messina, E., Manachini, B., Cappello, S. & Quatrini, P. The gut microbiota of larvae of *Rhynchophorus ferrugineus* Oliver (Coleoptera: Curculionidae). *BMC Microbiol.* **14**, 136. 10.1186/1471-2180-14-136 (2014). 24884866 10.1186/1471-2180-14-136 PMC4060583

[7] Naveed, H., Andoh, V., Islam, W., Chen, L. & Chen, K. Sustainable Pest Management in Date Palm Ecosystems: Unveiling the Ecological Dynamics of Red Palm Weevil (Coleoptera: Curculionidae) Infestations. *Insects* **14**, 859. 10.3390/insects14110859 (2023). 37999058 10.3390/insects14110859 PMC10671898

[8] Faleiro, J. R., Ferry, M., Yaseen, T. & Al-Dobai, S. Overview of the gaps, challenges and prospects of red palm weevil management. *Arab J. Plant. Prot* **37** (2019).

[9] Hosang, M. L. A. et al. Advanced Strategies for Managing of Red Palm Weevil (*Rhynchophorus ferrugineus*): Innovative Technologies and Integrated Solutions. In *Science-Based Pest Management for a Sustainable and Resilient Coconut Sector* (eds Alouw, J. C.) Ch. 18, 291–303 (Springer Nature Switzerland, 2025). 10.1007/978-3-031-84266-5_18

[10] Bansemir, A., Blume, M., Schröder, S. & Lindequist, U. Screening of cultivated seaweeds for antibacterial activity against fish pathogenic bacteria. *Aquaculture* **252**, 79–84. 10.1016/j.aquaculture.2005.11.051 (2006).

[11] Mulatier, M. et al. Invasive brown algae (Sargassum spp.) as a potential source of biocontrol against *Aedes aegypti*. *Sci. Rep.* **14**, 21161. 10.1038/s41598-024-72243-z (2024). 39256502 10.1038/s41598-024-72243-z PMC11387777

[12] Holdt, S. L. & Kraan, S. Bioactive compounds in seaweed: functional food applications and legislation. *J. Appl. Phycol.* **23**, 543–597. 10.1007/s10811-010-9632-5 (2011).

[13] Saber, A., Moussa, S., Abdel-Rahim, E. & Cantonati, M. Insecticidal prospects of algal and cyanobacterial extracts against the cotton leafworm *Spodoptera littoralis*. *Vie et Milieu*. **68**, 199–212 (2018).

[14] Alfy, H., Ghareeb, R. Y., El-Sabrout, A. M. & Malak, M. The Double Face Effect Of Algae Extracts As Bio-Pesticides Against Fall Armyworm, S*podoptera Frugiperda*, And Root-Knot Nematodes, *Meloidogyne Incognita*. *Int. J. Environ. Sci.* **11**, 862–879. 10.64252/0j1pv850 (2025).

[15] Asimakis, E. et al. Algae and Their Metabolites as Potential Bio-Pesticides. *Microorganisms* **10**10.3390/microorganisms10020307 (2022). 10.3390/microorganisms10020307 PMC8877611 35208762

[16] Beev, G. et al. Harnessing Marine Algae for Sustainable Agriculture: Natural Bioactive Compounds as Eco-Friendly Pesticidal Agents. *Mar. Drugs*. **23** (346). 10.3390/md23090346 (2025). 10.3390/md23090346 PMC12471970 41003315

[17] Hussni Hasan, N. R. et al. From the Sea to Mosquito Control: The Potential of *Halymenia dilatata* Marine Alga as an Eco-Friendly Mosquitocidal Agent. *Sustainability* **15**, 11900. 10.3390/su151511900 (2023).

[18] Refaay, D. A., El-Sheekh, M. M., Heikal, Y. M. & Rashed, A. A. Characterization of some selected macroalgae extracts and assessment of their insecticidal and genotoxicity in *Culex pipiens* L. mosquito larvae. *Sci. Rep.* **15**, 2655. 10.1038/s41598-025-86347-7 (2025). 39838058 10.1038/s41598-025-86347-7 PMC11751296

[19] El Shafay, S. M., Ali, S. S. & El-Sheekh, M. M. Antimicrobial activity of some seaweeds species from Red sea, against multidrug resistant bacteria. *Egypt. J. Aquat. Res.* **42**, 65–74. 10.1016/j.ejar.2015.11.006 (2016).

[20] El Semary, N. et al. Use of algae from an oasis in Saudi Arabia in production of biofuel and bio-fertilizer. *Bangladesh J. Bot.* **47**, 523–531. 10.3329/bjb.v47i3.38721 (2018).

[21] Aldawood, A. S. et al. Semi-artificial diet developed for the successful rearing of red palm weevil: *Rhynchophorus ferrugineus* (Coleoptera: Dryophthoridae) in the laboratory. *J. King Saud Univ. Sci.* **34**, 102272. 10.1016/j.jksus.2022.102272 (2022).

[22] El-Shafie, H., Faleiro, J., Abo-El-Saad, M. & Aleid, S. A meridic diet for laboratory rearing of red palm weevil, *Rhynchophorus ferrugineus* (Coleoptera: Curculionidae). *Sci. Res. Essays*. **8**, 1924–1932. 10.5897/SRE2013.5502 (2013).

[23] Hyötyläinen, T. & Riekkola, M. L. Direct coupling of reversed-phase liquid chromatography to gas chromatography. *J. Chromatogr. A*. **819**, 13–24. 10.1016/S0021-9673(98)00539-1 (1998).

[24] Agatonovic-Kustrin, S., Morton, D. W. & Ristivojević, P. Assessment of antioxidant activity in Victorian marine algal extracts using high performance thin-layer chromatography and multivariate analysis. *J. Chromatogr. A*. **1468**, 228–235. 10.1016/j.chroma.2016.09.041 (2016). 27670751 10.1016/j.chroma.2016.09.041

[25] Huang, Y., Jiang, H., Mao, X. & Ci, F. Laminarin and laminarin oligosaccharides originating from brown algae: preparation, biological activities, and potential applications. *J. Ocean. Univ. China*. **20**, 641–653. 10.1007/s11802-021-4584-8 (2021).

[26] Abdelhamid, A. A., Salama, K. S. M., Elsayed, A. M. & Gad, M. A. Ali Ali El-Remaily, M. Synthesis and Toxicological Effect of Some New Pyrrole Derivatives as Prospective Insecticidal Agents against the Cotton Leafworm, Spodoptera littoralis (Boisduval). *ACS Omega*. **7**, 3990–4000. 10.1021/acsomega.1c05049 (2022). 35155894 10.1021/acsomega.1c05049 PMC8829954

[27] Barrett, M. et al. Larval mannitol diets increase mortality, prolong development and decrease adult body sizes in fruit flies (*Drosophila melanogaster*). *Biol. Open.* **8**10.1242/bio.047084 (2020). 10.1242/bio.047084 PMC6955208 31822472

[28] Kikuta, S. Deployment of an attractive toxic sugar bait system (ATSB) with insecticide, for adult *Tribolium castaneum* (Coleoptera: Tenebrionidae). *J. Stored Prod. Res.* **83**, 97–102. 10.1016/j.jspr.2019.06.009 (2019).

[29] Ibrahim, K., Moustafa, Z. K., Ahmed, A. A. & Zyaan, O. Natural Larvicidal Agents: Chemical Composition and Toxicological Evaluation of Sidr Oil and Leaf Extracts Against Aquatic Common House Mosquito Larvae. *Egypt. J. Aquat. Biol. Fish.* **29**, 3363–3390. 10.21608/ejabf.2025.427611.6662 (2025).

[30] Essa, E., Abu El-Hassan, G. & Farag, S. Biochemical Composition, Toxicity and Bioactivities of the Essential Oil extracted from *Coffea arabica L.* husks against the Cotton Leafworm, *Spodoptera littoralis* (Boisduval) (Lepidoptera: Noctudiae). *Egypt. Acad. J. Biol. Sci. A Entomol* 15, 37–49 (2022). 10.21608/eajbsa.2022.254995

[31] Ramachandran, K. et al. Novel insecticidal properties of bioactive zoochemicals extracted from sea urchin *Salmacis virgulata*. *Open. Chem.* **23**10.1515/chem-2025-0141 (2025).

[32] Karthi, S. et al. Larvicidal Enzyme Inhibition and Repellent Activity of Red Mangrove *Rhizophora mucronata* (Lam.) Leaf Extracts and Their Biomolecules Against Three Medically Challenging Arthropod Vectors. *Molecules* **25**, 3844. 10.3390/molecules25173844 (2020). 32847069 10.3390/molecules25173844 PMC7504580

[33] Dawoud, S., Al-Akra, T. & Zedan, A. Antioxidant Activity of Some Natural Compounds in Alleviating the Hepatotoxicity Effects Induced by Emamectin Benzoate in Male Mice. *J. Agric. Chem. Biotechnol.* **12**, 145–156. 10.21608/jacb.2021.86457.1013 (2021).

[34] El-Aziz, F. E. Z. A. A. et al. Insecticidal activity of brown seaweed (*Sargassum latifolium*) extract as potential chitin synthase inhibitors: Toxicokinetic and molecular docking approaches. *S Afr. J. Bot.* **160**, 645–656. 10.1016/j.sajb.2023.07.058 (2023).

[35] Gliszczynska, A. et al. Synthesis of novel phytol-derived gamma-butyrolactones and evaluation of their biological activity. *Sci. Rep.* **11**, 4262. 10.1038/s41598-021-83736-6 (2021). 33608591 10.1038/s41598-021-83736-6 PMC7896091

[36] Mohy, E., Din, S. & Elahwany, A. Bioactivity and Phytochemical Constituents of Marine Red Seaweeds (Jania rubens, Corallina mediterranea and Pterocladia capillacea). *J. Taibah Univ. Sci.* **10**10.1016/j.jtusci.2015.06.004 (2015).

[37] Khalil, M. et al. Insecticidal and Repellent Activity of Essential Oils from Seven Different Plant Species against Tribolium castaneum (Coleoptera: Tenebrionidae). *Insects* **15**, 755. 10.3390/insects15100755 (2024). 39452331 10.3390/insects15100755 PMC11508915

[38] Okudan, E. & Eren, G. Insecticidal activity of forty-seven marine algae species from the Mediterranean, Aegean, and Sea of Marmara in connection with their cholinesterase and tyrosinase inhibitory activity. *S Afr. J. Bot.* **143**10.1016/j.sajb.2021.06.038 (2021).

[39] Yang, R., Lv, M. & Xu, H. Synthesis of Piperine Analogs Containing Isoxazoline/Pyrazoline Scaffold and Their Pesticidal Bioactivities. *J. Agric. Food Chem.* **66**, 11254–11264. 10.1021/acs.jafc.8b03690 (2018). 30295024 10.1021/acs.jafc.8b03690

[40] Varghese, J. A., Vendan, S. E., Rajashekar, Y. & Shivaramu, M. S. Sustainable insect pest management using bioactive compounds derived from marine macroalgae: A comprehensive review. *Bioresour Technol.* **455**, 134672. 10.1016/j.biortech.2026.134672 (2026). 42035956 10.1016/j.biortech.2026.134672

[41] Thitame, S. N. & Aher, A. A. Unveiling the Potential of Algal Secondary Metabolites in Natural Mosquito Repellence: A Review. *J. Pharm. Bioallied Sci.* **17**, 40–42. 10.4103/jpbs.jpbs_116_25 (2025). 10.4103/jpbs.jpbs_116_25 PMC12156503 40511162

[42] Magalhaes Xavier, K. C. et al. Phytochemical, cytotoxic, and insecticidal effects of crude extracts of the alga Alsidium triquetrum (SGGmelin) Trevisan on Aedes aegypti. *Biocatal. Agric. Biotechnol.* **63**, 103461. 10.1016/j.bcab.2024.103461 (2025).

[43] Pavia, D., Lampman, G., Kriz, G. & Vyvyan, J. Introduction to spectroscopy. *Cengage learning* (2015).

[44] Gheda, S. et al. Potent Effect of Phlorotannins Derived from *Sargassum linifolium* as Antioxidant and Antidiabetic in a Streptozotocin-Induced Diabetic Rats Model. *Appl. Sci.* **13**, 4711. 10.3390/app13084711 (2023).

[45] Rivera-Tovar, P. R. et al. Sustainable Recovery of Phlorotannins from *Durvillaea incurvata*: Integrated Extraction and Purification with Advanced Characterization. *Antioxidants* **14**, 250. 10.3390/antiox14030250 (2025). 40227225 10.3390/antiox14030250 PMC11939385

[46] Gómez-Ordóñez, E. & Rupérez, P. FTIR-ATR spectroscopy as a tool for polysaccharide identification in edible brown and red seaweeds. *Food Hydrocoll.* **25**, 1514–1520. 10.1016/j.foodhyd.2011.02.009 (2011).

[47] Darrag, H. M., Alhajhoj, M. R. & Khalil, H. E. Bio-Insecticide of *Thymus vulgaris* and *Ocimum basilicum* Extract from Cell Suspensions and Their Inhibitory Effect against Serine, Cysteine, and Metalloproteinases of the Red Palm Weevil (*Rhynchophorus ferrugineus*). *Insects* 12, 405 (2021). 10.3390/insects12050405 10.3390/insects12050405 PMC8147177 33946503

[48] Alyahya, H. S. Efficacy of aqueous and alcoholic formulations extracted from Padina boryana algae against *Aedes aegypti* mosquito larvae: A comparative study. *Entomol. Res.* **54**, e12748 (2024). 10.1111/1748-5967.12748.

[49] Perumal, P., Dhanasundaram, S., Aravinth, A., Amutha, V. & Santhanam, P. Larvicidal property of the extracts of the seaweeds; Sargassum wightii, S. ilicifolium and Gelidiella acerosa against *Anopheles stephensi*, *Aedes aegypti* and *Culex quinquefasciatus*. *Biocatal. Agric. Biotechnol.* **43**, 102436. 10.1016/j.bcab.2022.102436 (2022).

[50] Poornasundari, B., Arivoli, S., Dinakararajan, P., Samykannu, M. & Kumaresan, V. Investigation of Larvicidal Activity and Histopathological Variations of Brown Algae *Spatoglossum asperum J. Agardh* against Aedes aegypti, Culex quinquefasciatus, and Anopheles stephensi. *Sci. Technol. Asia*. **28**, 237–255 (2023). https://ph02.tci-thaijo.org/index.php/SciTechAsia/article/view/250181

[51] Jabbour, C. et al. Insecticidal Activity of Eco-Extracted Holopelagic Sargassum Against the Whitefly Bemisia tabaci Infesting Tomato Crops. *Phycology* **5**, 79. 10.3390/phycology5040079 (2025).

[52] Sohrabi, F. et al. Insecticidal efficacy of synthesized ZnO nanoparticles using brown algae Cystoseira baccata extract against Callosobruchus Maculatus (F.) (Col.: Chrysomelidae). (2024). 10.21203/rs.3.rs-3834522/v1

[53] Divekar, P. A. et al. Plant Secondary Metabolites as Defense Tools against Herbivores for Sustainable Crop Protection. *Int. J. Mol. Sci.* **23**10.3390/ijms23052690 (2022). 10.3390/ijms23052690 PMC8910576 35269836

[54] Grover, S. et al. Dynamic regulation of phenylpropanoid pathway metabolites in modulating sorghum defense against fall armyworm. *Front. Plant. Sci.* **13**, 1019266. 10.3389/fpls.2022.1019266 (2022). 36507437 10.3389/fpls.2022.1019266 PMC9732255

[55] Wijesekara, I., Pangestuti, R. & Kim, S. Biological activities and potential health benefits of sulfated polysaccharides derived from marine algae. *Carbohydr. Polym.* **11**, 14–21. 10.1016/J.CARBPOL.2010.10.062 (2010).

[56] Song, Y., Villeneuve, D. L., Toyota, K., Iguchi, T. & Tollefsen, K. E. Ecdysone Receptor Agonism Leading to Lethal Molting Disruption in Arthropods: Review and Adverse Outcome Pathway Development. *Environ. Sci. Technol.* **51**, 4142–4157. 10.1021/acs.est.7b00480 (2017). 28355071 10.1021/acs.est.7b00480 PMC6135102

[57] Osman, N., Abd-Elazeem, O. M., Al-Eisa, R. A. & El-Shenawy, N. S. Anticancer and antimicrobial evaluation of extract from brown algae *Hormophysa cuneiformis*. *J. Appl. Biomed.* **21**, 121–136. 10.32725/jab.2023.016 (2023). 37747312 10.32725/jab.2023.016

[58] Azzaz, N., Hamed, S. & El-Said, E. Abd El-ghany, S. Evaluation of Proximate Composition and Antioxidant Activities for Some Extracts of *Hormophysa cuneiformis*. *Damietta J. Agric. Sci.* **4**, 136–142. 10.21608/djas.2025.430277 (2025).

[59] Salem, W. M. Screening for antibacterial activities in some marine algae from the red sea (Hurghada, Egypt). *Afr. J. Microbiol. Res.* **5**, 2160–2167. 10.5897/ajmr11.390 (2011).

[60] Mohamed, S. & Saber, A. Antifungal potential of the bioactive constituents in extracts of the mostly untapped brown seaweed *Hormophysa cuneiformis* from the Egyptian coastal waters. *Egypt. J. Bot.* **59**, 695–708. 10.21608/EJBO.2019.5516.1225 (2019).

[61] Wu, S. et al. Research Progress of Marine Anti-Fouling Coatings. *Egypt. J. Bot.* **14**, 1227. 10.21608/EJBO.2019.5516.1225 (2024).

[62] Berteau, O. & Mulloy, B. Sulfated fucans, fresh perspectives: structures, functions, and biological properties of sulfated fucans and an overview of enzymes active toward this class of polysaccharide. *Glycobiology* **13**, 29–40. 10.1093/glycob/cwg058 (2003). 10.1093/glycob/cwg058 12626402

[63] Zhao, Y. et al. Fucoidan Extracted from Undaria pinnatifida: Source for Nutraceuticals/Functional Foods. *Mar. Drugs*. **16**10.3390/md16090321 (2018). 10.3390/md16090321 PMC6164441 30205616

[64] Aravinth, A. et al. Evaluation of Brown and red seaweeds-extracts as a novel larvicidal agent against the deadly human diseases-vectors, *Anopheles stephensi*, *Aedes aegypti* and *Culex quinquefasciatus*. *Exp. Parasitol.* **256**, 108651. 10.1016/j.exppara.2023.108651 (2024). 37944660 10.1016/j.exppara.2023.108651

[65] Elbrense, H. et al. Antifeedant and repellent efficacy of certain essential oils against adult rust-red flour beetle, Tribolium castaneum. *Egypt. J. Chem.* **65**, 167–178. 10.21608/ejchem.2021.79263.3897 (2021).

[66] Sterner, M. & Edlund, U. Multicomponent fractionation of *Saccharina latissima* brown algae using chelating salt solutions. *J. Appl. Phycol.* **28**, 2561–2574. 10.1007/s10811-015-0785-0 (2016). 27471344 10.1007/s10811-015-0785-0 PMC4947094

[67] Isman, M. B. Botanical Insecticides in the Twenty-First Century-Fulfilling Their Promise? *Annu. Rev. Entomol.* **65**, 233–249. 10.1146/annurev-ento-011019-025010 (2020). 31594414 10.1146/annurev-ento-011019-025010

[68] Gostin, I. N. & Popescu, I. E. Evaluation of the Essential Oils Used in the Production of Biopesticides: Assessing Their Toxicity toward Both Arthropod Target Species and Beneficial Pollinators. *Agriculture* **14**, 81. 10.3390/agriculture14010081 (2024).

[69] Ali, M. A., Mohanny, K. M., Mohamed, G. S. & Allam, R. O. H. Insecticidal potential of some plant extracts in nano and normal form on immatures stages of red palm weevil *Rhynchophorus ferrugineus*. *SVU Int. J. Agric. Sci.* **2**, 306–315. 10.21608/svuijas.2020.43031.1037 (2020).

[70] Ali, M. Y. S., Ravikumar, S. & Beula, J. M. Mosquito larvicidal activity of seaweeds extracts against *Anopheles stephensi*, *Aedes aegypti* and *Culex quinquefasciatus*. *Asian Pac. J. Trop. Dis.* **3**, 196–201. 10.1016/s2222-1808(13)60040-7 (2013).

[71] Yu, K. X., Wong, C. L., Ahmad, R. & Jantan, I. Mosquitocidal and Oviposition Repellent Activities of the Extracts of Seaweed Bryopsis pennata on *Aedes aegypti* and A*edes albopictus*. *Molecules* **20**, 14082–14102. 10.3390/molecules200814082 (2015). 26247928 10.3390/molecules200814082 PMC6332061

[72] Sekar, S., Jeyachandran, S., Giri, J. & Aman, M. Advancing sustainable agriculture: the potential of seaweed-derived bio pesticides from marine biomass. *Bioresour Bioprocess.* **12**, 22. 10.1186/s40643-025-00849-w (2025). 40120004 10.1186/s40643-025-00849-w PMC11929657

[73] Du, B. et al. A critical review on extraction, characteristics, physicochemical activities, potential health benefits, and industrial applications of fucoidan. *eFood* 3 (2022). 10.1002/efd2.19

[74] Jiao, W. et al. Effects of Molecular Weight and Guluronic Acid/Mannuronic Acid Ratio on the Rheological Behavior and Stabilizing Property of Sodium Alginate. *Molecules* **24**, 4374. 10.3390/molecules24234374 (2019). 31795396 10.3390/molecules24234374 PMC6930533

[75] Shrestha, S., Zhang, W., Smid, S. D. & Phlorotannins A review on biosynthesis, chemistry and bioactivity. *Food Biosci.* **39**, 100832. 10.1016/j.fbio.2020.100832 (2021).

[76] Steevensz, A. J. et al. Profiling phlorotannins in brown macroalgae by liquid chromatography-high resolution mass spectrometry. *Phytochem Anal.* **23**, 547–553. 10.1002/pca.2354 (2012). 22383068 10.1002/pca.2354

